# Gestational weight trajectory and risk of adverse pregnancy outcomes among women with gestational diabetes mellitus: A retrospective cohort study

**DOI:** 10.1111/mcn.13645

**Published:** 2024-03-22

**Authors:** Lihua Lin, Yanhong Huang, Lijuan Chen, Lianghui Zheng, Yebin Feng, Juan Lin, Jianying Yan

**Affiliations:** ^1^ Department of Healthcare, Fujian Maternity and Child Health Hospital Affiliated Hospital of Fujian Medical University Fuzhou People's Republic of China; ^2^ Department of Child Healthcare Center, Fujian Maternity and Child Health Hospital Affiliated Hospital of Fujian Medical University Fuzhou People's Republic of China; ^3^ Department of Obstetric, Fujian Maternity and Child Health Hospital Affiliated Hospital of Fujian Medical University Fuzhou People's Republic of China; ^4^ Department of Research Office, Fujian Maternity and Child Health Hospital Affiliated Hospital of Fujian Medical University Fuzhou People's Republic of China

**Keywords:** adverse pregnancy outcomes, body mass index, gestational diabetes mellitus, gestational weight gain, latent class trajectory model

## Abstract

The aim of this study was to explore gestational weight gain (GWG) trajectories and their associations with adverse pregnancy outcomes. A retrospective cohort study including 11,064 women with gestational diabetes mellitus (GDM) was conducted between 2015 and 2019 in China. The latent class trajectory model was used to identify GWG trajectories, and logistic regression was performed to examine odds ratio (OR) of pregnancy outcomes. Three trajectories of GWG were identified in these 11,604 women with GDM. Trajectory 1: 64.02% of women had sustained moderate GWG throughout pregnancy; Trajectory 2: 17.75% of women showed a high initial GWG but followed by a low GWG from the third trimester until delivery; Trajectory 3: 18.23% had low initial GWG but followed by drastic GWG from the second trimester until delivery. Compared with pregnant women with Trajectory 1, women with Trajectory 2 had a higher risk of large for gestational age (adjusted odds ratio [AOR]: 1.29, 95% confidence interval [CI]: 1.12–1.48) but at a lower risk of having hypertensive disorders of pregnancy (AOR: 0.76, 95% CI: 0.57–0.96). Women in Trajectory 3 were more likely to develop small for gestational age (AOR: 2.12, 95% CI: 1.62–2.78), low birthweight (AOR: 1.49, 95% CI: 1.07–2.08), preterm birth (AOR: 1.28, 95% CI: 1.05–1.63), caesarean section (AOR: 1.26, 95% CI: 1.112–1.42) and hypertensive disorders of pregnancy (AOR: 2.24, 95% CI: 1.82–2.76). The association of GWG trajectory with adverse pregnancy outcomes differs across prepregnancy body mass index and GWG categories. Women with a slow initial GWG but followed by drastic GWG had higher risks of adverse pregnancy outcomes. Early clinical recognition of poor GWG trajectory will contribute to early intervention in high‐risk groups to minimise adverse outcomes.

## Key messages

- Gestational weight gain (GWG) is a controllable factor and is strongly associated with maternal and fetal outcomes, especially among gestational diabetes mellitus.
- The same total GWG may present different trajectories, and the trajectory patterns of GWG vary with women in different body mass index categories.
- Clarifying the relationship between GWG trajectory and adverse pregnancy outcomes may contribute to the clear goal of lifestyle interventions based on weight control.
- Early recognition of an unhealthy GWG trajectory may contribute to early intervention in high‐risk groups to minimise adverse outcomes.

## Graphical abstract

Gestational weight gain was identified using the latent class trajectory model. Binary logistic regression was performed to examine the associations between adverse pregnancy outcomes and these trajectories.

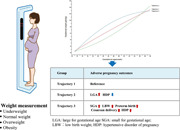

## 1 INTRODUCTION

Gestational diabetes mellitus (GDM) is one of the most common pregnancy complications, conferring tremendous short‐ or long‐term health consequences for both mothers and their children, such as caesarean section, hypertensive disorder of pregnancy (HDP), preeclampsia for mother and large for gestational age (LGA), shoulder dystocia and macrosomia for the infant (Buchanan et al., 2012; Khambalia et al., 2017; Reece et al., 2009). Women with GDM are more likely to develop type 2 diabetes mellitus and cardiovascular disease in the future (Juan & Yang, 2020; Juan et al., 2019). Offspring born to mothers with GDM have higher risk of obesity in childhood as well as cardiovascular disease in adulthood (American Diabetes Association, 2020). The prevalence of GDM increases rapidly and affects about 15% of pregnancies in China (Gao et al., 2019), posing a great threat to public health. Therefore, a focus on GDM management in China is of great importance. Good glycemic control has been shown to reduce complications in women with GDM (Horvath et al., 2010). In addition to blood glycemic control, optimal gestational weight gain (GWG) is an important goal. GWG is a controllable factor and is strongly associated with maternal and fetal outcomes, especially among GDM. Suboptimal GWG is a well‐known risk factor for adverse pregnancy outcomes. The Institute of Medicine Committee reexamined GWG guidelines in 2009 according to prepregnancy body mass index (BMI) for a singleton pregnancy and twin pregnancies, and these are often applied to special populations such as GDM (Barnes et al., 2020; Institute of Medicine, 2009). These guidelines were updated to lower the adverse pregnancy outcomes for both mothers and their infants. Emerging evidence has suggested higher prepregnancy BMI is linked with higher risk of poor maternal and neonatal consequences, such as LGA, HDP and caesarean section (Graham et al., 2014; Rasmussen et al., 2010), and when combined with GDM, showing as the most major determinant factor for macrosomia and LGA (Ricart et al., 2005; Ryan, 2011; Wang et al., 2016).

Limited research on GWG in patients with GDM is available. Previous studies on GWG issues mainly focus on the total GWG or the rate of GWG at special trimesters. However, there might be different patterns of GWG among a population with the same total GWG. Therefore, trajectory patterns of GWG should be given enough attention. Previous studies have shown trajectory patterns of GWG, which vary with women in different BMI categories (Jarman et al., 2016; Piccinini‐Vallis et al., 2016). The relationship between GWG trajectory patterns and adverse pregnancy outcomes in patients with GDM has been less studied. The traditional approach to GDM management largely focusses on lifestyle intervention to monitor and treat maternal hyperglycaemia. However, in addition to the physiological increase in weight gain during pregnancy, excessive weight gain could produce a higher insulin resistance, which could further exacerbate maternal hyperglycaemia (Bogdanet et al., 2018; Metzger, 2005; Oteng‐Ntim et al., 2018). Hence, if the relationship between GWG trajectory and adverse pregnancy outcomes is clarified, the goal of lifestyle interventions based on weight control could be clearer and improve adherence rates. Moreover, the GWG during different trimesters is not independent, which might be influenced by previous trimesters (Zheng et al., 2019). Thus, constructing and monitoring weight gain trajectories during pregnancy helps to comprehensively study GWG and identify high‐risk populations for targeted intervention. Most research on this issue mainly focus on the ‘optima’ total GWG or assess the relationship between total GWG and adverse pregnancy outcome, with little attention on the trajectory of GWG for GDM. Knowing the trajectory of GWG and its influence on pregnancy outcomes may contribute to developing early preventive measures that would benefit maternal and infant health. Therefore, we aimed to construct the trajectory patterns of GWG among women with GDM and further estimate the association between GWG trajectory and pregnancy outcomes stratified by prepregnancy BMI and total GWG categories.

## 2 METHODS

### 2.1 Study design and population

We carried out a retrospective observational cohort study of pregnant women with GDM who had received regular maternal health care and delivered a live singleton neonate at Fujian Maternity and Child Health Hospital from January 2015 to December 2019. The Ethics Committee of Fujian Maternity and Child Health Hospital approved this study. A total of 16,803 GDM medical records were screened, and women were excluded for the following reasons: maternal age less than 18 years, prepregnancy diabetes, prepregnancy hypertension, chronic heart disease, kidney disease, autoimmune disease, stillbirth, miscarriage, birth defect, with twin or multiple births and with missing data. The remaining 11,064 women were included in the final analysis.

### 2.2 Definitions and measurements

Clinical information including demographic data, obstetric data and delivery data was reviewed and extracted into a database for further sort and analysis. Demographic characteristics included maternal age, prepregnancy weight, height, education level (university or above, below university) and employment (employed, unemployed). Obstetrical information included gravidity (1, 2, ≥3), parity (primiparous or multiparous) and maternal weight at each perinatal visit. Delivery data included discharge diagnosis, mode of delivery, gestational age at delivery, infant sex, birthweight and birth length. Prepregnancy BMI was calculated as prepregnancy weight (kg)/height^2 ^(m^2^) and classified into four groups using Chinese standard: underweight (<18.5 kg/m^2^), normal weight (18.5–23.9 kg/m^2^), overweight (24.0–27.9 kg/m^2^) and obesity (≥28.0 kg/m^2^) (Zhou, 2002).

The prepregnancy weight was self‐reported by pregnant women at the first antenatal visit, and maternal weight was measured wearing light clothing and no shoes by trained nurses using a weighing scale (Inbody 270) at each following antenatal visit during pregnancy. The GWG was defined as the difference between maternal weight at each antenatal visit and prepregnancy weight, and the total GWG was the difference between the maternal weight at delivery and prepregnancy weight. Each pregnant woman usually receives 12–14 prenatal visits throughout the whole pregnancy. That is, monthly visits until 28 weeks' gestation, bimonthly visits until 36 weeks' gestation and weekly visits until delivery. Due to the irregularity of the first prenatal visit (before 13 gestational weeks), to ensure the reliability of weight gain trajectories, we restricted women to at least one‐time weight measurement in the first (before 13 gestation weeks), second (from 14 to 28 gestation weeks) and third (from 29 gestation weeks to delivery) pregnancy trimesters.

The total GWG was divided as ‘inadequate’, ‘adequate’ or ‘excessive’ in the Chinese recommendations according to BMI categories (Chinese Nutrition Society, 2021) (Table S1). The gestational age was ascertained by ultrasonography. GDM was diagnosed based on a 75 g oral glucose tolerance test at 24–28 weeks gestation when at least one of the following values was observed: fasting blood glucose level ≥5.1 mmol/L, a 1‐h blood glucose level ≥10.0 mmol/L and a 2‐h blood glucose level ≥8.5 mmol/L (Metzger et al., 2010).

The main adverse pregnancy outcomes in this study included macrosomia, LGA, small for gestational age (SGA), low birthweight (LBW), preterm birth, caesarean section and HDP. An infant's birthweight of more than 4000 g was defined as macrosomia. LGA or SGA refers to birthweight more than the 90th or less than the 10th percentile of a standardised birthweight based on gender and gestational age, respectively (Villar et al., 2014). LBW was defined as birthweight less than 2500 g. Preterm birth was considered as infants born before 37 weeks. HDP was defined as blood pressure ≥140/90 mmHg that occurred after 20 weeks gestation but without proteinuria (Fatima et al., 2021).

### 2.3 Statistical analyses

The latent class trajectory model (LCTM) was used to identify the weight gain patterns and trajectories of GDM women over the whole pregnancy time (Nagin et al., 2018). The LCTM is a special form of finite mixture modelling designed to identify potential classes of individuals based on the similar progression of determinants over time or age (Jones & Nagin, 2013). We first constructed a scoping model and then identified the optimal number of classes (testing *K* = 1–7) based on the lowest Bayesian information criterion values (Lennon et al., 2018). After that, we performed a model adequacy assessment by calculating the largest estimated probability of assignments of women being assigned to which trajectory class. The largest estimated probability of assignments above 70% is regarded as acceptable. After establishing the best fitting models and each women's belonging trajectory groups, we further explored the relationship of GWG trajectory groups and adverse pregnancy outcomes using univariate and multivariate binary logistic regression, adjusted for prepregnancy BMI, maternal age, educational level, gravity, parity, employment, infant sex, gestational age and oral glucose tolerance test values. Our previous study suggests that both prepregnancy overweight or obesity and excessive GWG contribute to adverse pregnancy outcomes independently among women with gestational diabetes mellitus, and the multiplicative interaction for adverse pregnancy outcomes was observed (Lin et al., 2022). Therefore, we carried out a subgroup analysis based on total GWG and prepregnancy BMI groups and explored the relationship between GWG trajectory groups and adverse pregnancy outcomes.

Continuous variables of maternal characteristics are shown as mean ± standard deviation (SD) or median (interquartile range, IQR), and categorical variables are shown as numbers (percentage). Data were compared using analysis of variance (ANOVA) or nonparametric tests for continuous variables and *χ* ^2^ analyses for categorical variables. All analyses were performed using R software (version 4.2.4). *p* < 0.05 was considered statistically different. Crude odds ratio (OR), adjusted OR (AOR) and 95% confidence interval (CI) were used to report the effect estimates.

### 2.4 Ethics approval and consent to participate

Ethical approval was obtained from the Fujian Maternity and Child Hospital Ethics Committee. All procedures involving human participants performed in the studies have been reviewed by the Fujian Maternity and Child Hospital Ethics Committee and were in accordance with the Helsinki Declaration. All the data were obtained from patient records and the Ethics Committee of Fujian Maternity and Child Health Hospital waived the informed consent.

## 3 RESULTS

### 3.1 Basic characteristics of the GWG trajectories

**Figure 1:**
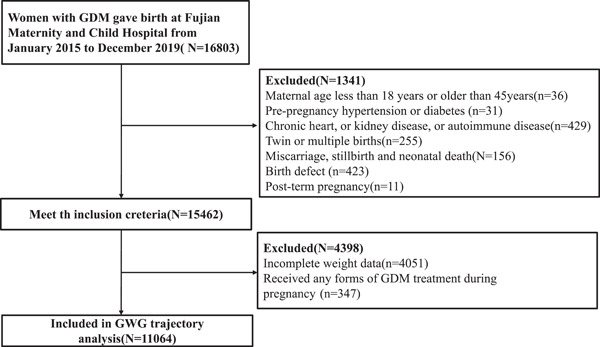
Flowchart of subject selection. GDM, gestational diabetes mellitus; GWG, gestational weight gain.

A total of 16,803 pregnant women with GDM receive prenatal examinations and delivery at Fujian Maternity and Child Hospital from January 2015 to December 2019. We excluded 5739 women from the following: maternal age less than 18 or more than 45 years (*n* = 36), miscarriage, stillbirth and neonatal death (*n* = 156), birth defect (*n* = 423), prepregnancy hypertension or diabetes (*n* = 31), chronic heart disease, or kidney disease, or autoimmune disease (*n* = 429), with twin or multiple births (*n* = 255), post‐term pregnancy (*n* = 11) and with incomplete weight data (*n* = 4051). After excluding women who received any forms of GDM treatment during pregnancy (*n* = 347), a sample of 11,064 GDM women was included in the final analysis (Figure 1). The whole times of weight measurements for all the study population was 129,184, with a median of 12 measurements (interquartile range, IQR 10–13), ranging from 5 to 21 times of measurements per woman. All the participants received the guideline to control weight gain at each antenatal visit from obstetrician, and 49.23% of the participants obeyed the guidelines.

**Figure 2:**
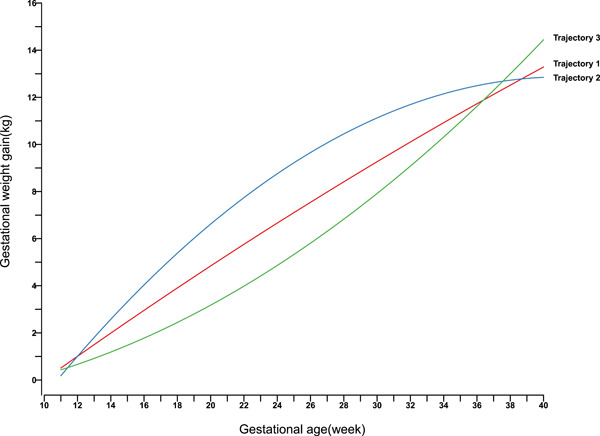
Gestational weight gain trajectory of women with gestational diabetes mellitus.

As the first prenatal visit is usually at about 11 gestational weeks, therefore, the GWG trajectory before 11 gestational weeks was not included in the trajectory analysis. Figure 2 shows the three trajectory patterns of GWG. The characteristics of the three trajectories are presented in Table 1. Trajectory 1 showed sustained moderate GWG throughout pregnancy, which accounted for 64.02% of the total study population. Trajectory 2 showed a high initial GWG but was followed by a low GWG from the third trimester until delivery, which was identified in 17.75% of the total sample population. Trajectory 3 had a low initial GWG but was followed by drastic GWG from the second trimester until delivery, comprised 18.23% of the total sample population. In the Trajectory 1 group, the median values of GWG in the first, second and third trimesters were 1.0, 6.5 and 5.6 kg, respectively. The GWG increased from 1.6 kg in the first trimester to 8.2 kg in the second trimester and 3.9 kg in the third trimester in Trajectory 2. The GWG in Trajectory 3 increased from 0.8 kg in the first trimester to 5.4 kg in the second trimester and 7.5 kg in the third trimester.

**Table 1:** Basic characteristics of the study population by the trajectory of gestational weight gain among women with gestational diabetes mellitus.

Variable	Overall (*n* = 11,064)	Trajectory of gestational weight gain			*p* Value
		Trajectory 1 (*n* = 7083, 64.02%)	Trajectory 2 (*n* = 1964, 17.75%)	Trajectory 3 (*n* = 2017, 18.23%)	
Maternal age (mean ± SD), years	31.2 ± 4.6	31.3 ± 4.6	31.6 ± 4.9	30.3 ± 4.4	<0.001, 1 vs. 2, 1 vs. 3, 2 vs. 3
Educational level, *n* (%)					<0.001, 1 vs. 2, 2 vs. 3
University degree or above	2614 (23.63)	1730 (24.4)	400 (20.4)	484 (24)	
Below university degree	8450 (76.37)	5353 (75.6)	1564 (79.6)	1533 (76)	
Employment, n (%)					0.522
Employed	6428 (58.10)	4141 (58.5)	1121 (57.1)	1166 (57.8)	
Unemployed	4636 (41.90)	2942 (41.5)	843 (42.9)	851 (42.2)	
Prepregnancy weight (mean ± SD), kg	55.24 ± 8.42	55.0 ± 8.3	55.1 ± 7.6	56.2 ± 9.6	<0.001, 1 vs. 3, 2 vs. 3
Prepregnancy BMI, *n* (%)					<0.001, 1 vs. 2, 1 vs. 3, 2 vs. 3
Underweight	1493 (13.49)	971 (13.7)	223 (11.4)	299 (14.8)	
Normal weight	7410 (66.97)	4768 (67.3)	1403 (71.4)	1239 (61.4)	
Overweight and obese	2161 (19.54)	1107 (15.6)	297 (15.1)	356 (17.6)	
Gravidity, *n* (%)					<0.001, 1 vs. 3
1	3849 (34.8)	2367 (33.4)	692 (35.2)	790 (39.2)	
2	3293 (29.8)	2149 (30.3)	552 (28.1)	592 (29.4)	
≥3	3922 (35.4)	2567 (36.2)	720 (36.7)	635 (31.5)	
Parity, *n* (%)					<0.001, 1 vs. 2, 1 vs. 3
Primipara	5712 (51.6)	3547 (50.1)	1056 (53.8)	1109 (55)	
Multipara	5352 (48.4)	3536 (49.9)	908 (46.2)	908 (45)	
Gestational weight gain value					
Total gestational weight gain (mean ± SD), kg	12.7 ± 4.4	12.7 ± 4.3	12.5 ± 4.6	13.1 ± 4.8	<0.001, 1 vs. 3, 2 vs. 3
First trimester gestational weight gain (median [IQR]), kg	1.0 (0.0, 2.5)	1.0 (0.0, 2.50)	1.6 (0.1, 2.90)	0.8 (0.0, 2.0)	<0.001, 1 vs. 2, 1 vs. 3, 2 vs. 3
Second trimester gestational weight gain (median [IQR]), kg	6.6 (4.9, 8.3)	6.5 (5.0, 8.1)	8.2 (6.7, 10.0)	5.4 (3.7, 7.0)	<0.001, 1 vs. 2 and 3, 2 vs. 3
Third trimester gestational weight gain (median [IQR]), kg	5.7 (3.7, 8.0)	5.6 (3.8, 7.6)	3.9 (1.8, 5.9)	7.5 (5.5, 9.6)	<0.001, 1 vs. 2, 1 vs. 3, 2 vs. 3
Gestational weight gain categories, *n* (%)					<0.001, 1 vs. 3, 2 vs. 3
Inadequate	1443 (13.04)	949 (13.4)	258 (13.1)	236 (11.7)	
Adequate	5447 (49.23)	3581 (50.6)	975 (49.6)	891 (44.2)	
Excessive	4174 (37.73)	2553 (36)	731 (37.2)	890 (44.1)	
Gestational age (mean ± SD), weeks	38.6 ± 1.5	38.6 ± 1.6	38.6 ± 1.5	38.6 ± 1.5	0.776
Oral glucose tolerance test					
Fasting plasma glucose level (mmol/L)	5.6 ± 3.1	5.5 ± 3.1	5.7 ± 2.9	5.5 ± 3.2	0.126
1 h plasma glucose level (mmol/L)	9.6 ± 3.5	9.7 ± 3.5	9.7 ± 3.6	9.4 ± 3.6	0.051
2 h plasma glucose level (mmol/L)	8.0 ± 3.0	8.0 ± 3.0	8.0 ± 3.1	7.9 ± 3.1	0.440
Caesarean section	4159 (37.59)	2623 (37.0)	739 (37.6)	797 (39.5)	0.127
Birth weight (mean ± SD), g	3286.0 ± 474.4	3284.2 ± 474.9	3337.3 ± 461.2	3242.3 ± 480.9	<0.001, 1 vs. 2 and 3, 2 vs. 3
Birth length (mean ± SD), cm	49.5 ± 2.0	49.5 ± 2.0	49.7 ± 1.9	49.3 ± 2.0	<0.001, 1 vs. 2, 1 vs. 3, 2 vs. 3
Infant sex, *n* (%)					0.002, 1 vs. 2, 2 vs. 3
Boy	5984 (54.1)	3783 (53.4)	1132 (57.6)	1069 (53)	
Girl	5080 (45.9)	3300 (46.6)	832 (42.4)	948 (47)	

*Note*: Data are shown as mean ± SD or median (IQR) for continuous variables and *n* (%) for categorical variables. Trajectory 1, 2, 3: patterns of gestational weight gain.

Abbreviations: BMI, body mass index; IQR, interquartile range; SD, standard deviation.

Maternal age, education level, first trimester GWG, second trimester GWG, third trimester GWG, birthweight and birth length differ between any two trajectory groups (*p* < 0.001). The distribution of prepregnancy BMI categories was significantly different among the three trajectories. Among the 7083 (64.02%) women in Trajectory 1, 67.3% had a normal weight BMI, while 11.4% of the 1964 (17.75%) women in Trajectory 2 were underweight, and 17.6% of 2017 (18.23%) women in Trajectory 3 were overweight and obese. Women of Trajectory 3 gained more total GWG significantly than the other two trajectories (*p* < 0.001) and more proportion in excessive weight gain (*p* < 0.001). No significant differences were found in education level, gestational age, fasting plasma glucose, 1 h plasma glucose and 2 h plasma glucose across the three GWG trajectories.

### 3.2 Associations of GWG trajectory with adverse pregnancy outcomes

Table 2 shows the ORs (95% CI) of suffering adverse pregnancy outcomes for women in Trajectory 2 and 3, compared with women in Trajectory 1, that is, ‘sustained moderately GWG throughout pregnancy’ was used as the reference group. After adjusting for prepregnancy BMI, maternal age, educational level, gravity, parity, employment, infant sex, gestational age and oral glucose tolerance test values, women in Trajectory 2 had a higher risk of LGA (AOR: 1.29, 95% CI: 1.12–1.48) but a lower risk of HDP (AOR: 0.76, 95% CI: 0.57–0.96). Women in Trajectory 3 were more likely to develop SGA (AOR: 2.12, 95% CI: 1.62–2.78), LBW (AOR: 1.49, 95% CI: 1.07–2.08), preterm birth (AOR: 1.28, 95% CI: 1.05–1.63), caesarean section (AOR: 1.26, 95% CI: 1.12–1.42) and HDP (AOR: 2.24, 95% CI: 1.82–2.76), compared with women in Trajectory 1.

**Table 2:** Association of the trajectory of gestational weight gain with adverse pregnancy outcomes.

Outcomes	Total	n. event_%	Crude OR (95% CI)	*p*_1_ Value	Adjusted OR (95% CI)	*p*_2_ Value
Macrosomia						
Trajectory 1	7083	388 (5.5)	1.00 (Ref)		1.00 (Ref)	
Trajectory 2	1964	123 (6.3)	1.15 (0.94–1.42)	0.183	1.12 (0.87–1.43)	0.378
Trajectory 3	2017	91 (4.5)	0.82 (0.65–1.03)	0.087	0.75 (0.57–1.01)	0.058
LGA						
Trajectory 1	7083	1376 (19.4)	1.00 (Ref)		1.00 (Ref)	
Trajectory 2	1964	461 (23.5)	1.27 (1.13–1.43)	<0.001	1.29 (1.12–1.48)	<0.001
Trajectory 3	2017	352 (17.5)	0.88 (0.77–0.99)	0.046	0.86 (0.784–1.01)	0.051
SGA						
Trajectory 1	7083	212 (3.0)	1.00 (Ref)		1.00 (Ref)	
Trajectory 2	1964	53 (2.7)	0.90 (0.66–1.22)	0.494	0.88 (0.62–1.27)	0.496
Trajectory 3	2017	105 (5.2)	1.78 (1.4–2.26)	<0.001	2.12 (1.62–2.78)	<0.001
LBW						
Trajectory 1	7083	330 (4.7)	1.00 (Ref)		1.00 (Ref)	
Trajectory 2	1964	81 (4.1)	0.88 (0.69–1.13)	0.314	0.82 (0.56–1.22)	0.335
Trajectory 3	2017	120 (5.9)	1.29 (1.04–1.61)	0.019	1.49 (1.07–2.08)	0.018
Preterm birth^a^						
Trajectory 1	7083	494 (7.0)	1.00 (Ref)		1.00 (Ref)	
Trajectory 2	1964	142 (7.2)	1.04 (0.86–1.26)	0.695	1.12 (0.87–1.35)	0.278
Trajectory 3	2017	157 (7.8)	1.13 (0.93–1.36)	0.214	1.28 (1.05–1.63)	<0.001
Caesarean section						
Trajectory 1	7083	2623 (37)	1.00 (Ref)		1.00 (Ref)	
Trajectory 2	1964	739 (37.6)	1.03 (0.93–1.14)	0.629	0.97 (0.86–1.10)	0.670
Trajectory 3	2017	797 (39.5)	1.11 (1.01–1.23)	0.042	1.26 (1.12–1.42)	<0.001
HDP						
Trajectory 1	7083	370 (5.2)	1.00 (Ref)		1.00 (Ref)	
Trajectory 2	1964	77 (3.9)	0.74 (0.58–0.95)	0.019	0.76 (0.57–0.96)	0.006
Trajectory 3	2017	218 (10.8)	2.2 (1.85–2.62)	<0.001	2.24 (1.82–2.76)	<0.001

*Note*: Trajectory 1, 2, 3: patterns of gestational weight gain.

*p*_1_ value: not adjustment.

*p*_2_ value: For all outcomes except preterm birth were adjusted for prepregnany body mass index, maternal age, educational level, gravity, parity, employment, infant sex, gestational age and oral glucose tolerance test values.

Abbreviations: CI, confidence interval; HDP, hypertensive disorders of pregnancy; LGA, large for gestational age; OR, odds ratio; SGA, small for gestational age.

^a^ Adjusted for prepregnany body mass index, maternal age, educational level, gravity, parity, employment, infant sex and oral glucose tolerance test values.

### 3.3 Subgroup analysis

Table 3 presents the risk of developing adverse pregnancy outcomes in the three trajectory groups stratified by prepregnancy BMI and total GWG categories. Compared with women in Trajectory 1, there was no significant difference between the risk of adverse pregnancy outcomes in the Trajectory 2 group among underweight and overweight and obese women but had a higher risk of LGA (AOR: 1.28, 95% CI: 1.08–1.51) among normal weight women. The association of GWG Trajectory 3 with adverse pregnancy outcomes differs across prepregnancy BMI categories. Underweight women with Trajectory 3 were associated with a higher risk of SGA (AOR: 2.90, 95% CI: 1.60–5.27); normal weight women with Trajectory 3 showed a higher risk of SGA (AOR: 1.89, 95% CI: 1.34–2.68), LBW (AOR: 1.70, 95% CI: 1.13–2.57), preterm birth (AOR: 1.43, 95% CI: 1.12–1.89), caesarean section (AOR: 1.26, 95% CI: 1.08–1.46) and HDP (AOR: 2.73, 95% CI: 2.10–3.54) but lower risk of macrosomia (AOR: 0.61, 95% CI: 0.41–0.91) and LGA (AOR: 0.83, 95% CI: 0.69–0.98). Moreover, overweight& obese women with Trajectory 3 increased the risk of SGA (AOR: 2.07, 95% CI: 1.06–4.02), caesarean section (AOR: 1.37, 95% CI: 1.08–1.76) and HDP (AOR: 2.83, 95% CI: 1.28–2.61).

**Table 3:** Subgroup analysis of the association between gestational weight gain trajectories and risk of adverse pregnancy outcomes by prepregnancy body mass index (BMI) and total gestational weight gain (GWG).^a^

Category	Macrosomia	LGA	SGA	LBW	Preterm birth^b^	Caesarean section	HDP
Prepregnancy BMI							
Underweight							
Trajectory 1	1.00 (Ref)	1.00 (Ref)	1.00 (Ref)	1.00 (Ref)	1.00 (Ref)	1.00 (Ref)	1.00 (Ref)
Trajectory 2	0.83 (0.26–2.68)	1.05 (0.63–1.75)	1.34 (0.61–2.93)	0.70 (0.21–2.38)	1.48 (0.77–2.83)	0.87 (0.58–1.30)	0.51 (0.11–2.28)
Trajectory 3	1.62 (0.66–3.96)	0.92 (0.57–1.49)	2.90 (1.60–5.27)	1.17 (0.46–2.96)	1.16 (0.86–1.62)	1.18 (0.83–1.66)	0.90 (0.29–2.83)
Normal weight							
Trajectory 1	1.00 (Ref)	1.00 (Ref)	1.00 (Ref)	1.00 (Ref)	1.00 (Ref)	1.00 (Ref)	1.00 (Ref)
Trajectory 2	1.20 (0.89–1.62)	1.28 (1.08–1.51)	0.74 (0.47–1.16)	0.79 (0.49–1.25)	1.29 (0.87–1.71)	0.92 (0.80–1.07)	0.60 (0.41–0.89)
Trajectory 3	0.61 (0.41–0.91)	0.83 (0.69–0.98)	1.89 (1.34–2.68)	1.70 (1.13–2.57)	1.43 (1.12–1.89)	1.26 (1.08–1.46)	2.73 (2.10–3.54)
Overweight or obese							
Trajectory 1	1.00 (Ref)	1.00 (Ref)	1.00 (Ref)	1.00 (Ref)	1.00 (Ref)	1.00 (Ref)	1.00 (Ref)
Trajectory 2	0.92 (0.57–1.50)	1.37 (0.97–1.86)	1.19 (0.47–3.03)	0.98 (0.34–2.80)	0.72 (0.42–1.26)	1.18 (0.89–1.57)	1.12 (0.70–1.79)
Trajectory 3	0.89 (0.58–1.37)	0.93 (0.70–1.23)	2.07 (1.06–4.02)	1.21 (0.56–2.58)	1.07 (0.68–1.62)	1.37 (1.08–1.76)	1.83 (1.28–2.61)
Total GWG							
Inadequate							
Trajectory 1	1.00 (Ref)	1.00 (Ref)	1.00 (Ref)	1.00 (Ref)	1.00 (Ref)	1.00 (Ref)	1.00 (Ref)
Trajectory 2	0.64 (0.18–2.29)	1.33 (0.79–2.21)	0.18 (0.04–0.76)	0.39 (0.11–1.36)	0.74 (0.41–1.37)	0.92 (0.65–1.32)	0.93 (0.41–2.09)
Trajectory 3	0.32 (0.07–1.41)	0.86 (0.47–1.57)	1.29 (0.65–2.57)	0.68 (0.26–1.79)	2.07 (1.28–3.34)	1.30 (0.91–1.86)	1.87 (0.99–3.55)
Adequate							
Trajectory 1	1.00 (Ref)	1.00 (Ref)	1.00 (Ref)	1.00 (Ref)	1.00 (Ref)	1.00 (Ref)	1.00 (Ref)
Trajectory 2	1.22 (0.83–1.81)	1.30 (1.06–1.60)	1.43 (0.92–2.21)	1.03 (0.62–1.71)	1.04 (0.76–1.43)	0.85 (0.71–1.01)	0.68 (0.43–1.08)
Trajectory 3	0.88 (0.56–1.38)	0.83 (0.65–1.05)	2.03 (1.35–3.06)	1.62 (1.01–2.59)	1.12 (0.80–1.55)	1.15 (0.96–1.38)	1.92 (1.36–2.71)
Excessive							
Trajectory 1	1.00 (Ref)	1.00 (Ref)	1.00 (Ref)	1.00 (Ref)	1.00 (Ref)	1.00 (Ref)	1.00 (Ref)
Trajectory 2	1.11 (0.79–1.55)	1.28 (1.03–1.59)	0.54 (0.24–1.22)	0.80 (0.37–1.72)	1.47 (0.99–2.17)	1.16 (0.95–1.42)	0.76 (0.49–1.17)
Trajectory 3	0.66 (0.46–0.95)	0.80 (0.65–0.99)	3.03 (1.93–4.75)	2.14 (1.20–3.83)	1.20 (0.83–1.75)	1.32 (1.10–1.58)	2.47 (1.85–3.30)

*Note*: Data are shown as OR (95% CI).

Trajectory 1, 2, 3: patterns of gestational weight gain.

Abbreviations: CI, confidence interval; HDP, hypertensive disorders of pregnancy; LGA, large gestational age; OR, odds ratio; SGA, small gestational age.

^a^ For all outcomes except preterm birth were adjusted for prepregnany body mass index, maternal age, educational level, gravity, parity, employment, infant sex, gestational age and oral glucose tolerance test values.

^b^ Adjusted for prepregnany body mass index, maternal age, educational level, gravity, parity, employment, infant sex and oral glucose tolerance test values.

The effect of total GWG on the association between GWG trajectory and pregnancy outcomes shows that GDM women gain weight following Trajectory 3 will increased risk of adverse pregnancy outcomes, especially if the total GWG exceeds the recommendations. In addition, GDM women gained adequate or excessive weight during pregnancy following Trajectory 2 have a higher risk of LGA.

## 4 DISCUSSION

To our best knowledge, it is the first study to identify the trajectories of GWG among women with GDM based on a retrospective cohort in Fujian, China. We identified three GWG trajectories using the latent class trajectory model among women with GDM and explored their associations with the risk of related adverse pregnancy outcomes. The percentages of three trajectories were 64.02%, 17.75% and 18.23%, respectively, in the study, which was not consistent with those among general pregnant women (Lin et al., 2023). The possible reason of the difference might be that for GDM pregnant women, they were asked to control weight gain during the third trimester. Trajectory 1 showed sustained moderate GWG throughout pregnancy and accounted for the majority of the sample population. Trajectory 2 presented a high initial GWG but then followed a low GWG, with a median GWG of 1.6 kg in the first trimester, 8.2 kg in the second trimester and 3.9 kg in the third trimester. Trajectory 3 with lower GWG in early pregnancy and relatively higher GWG in mid and late pregnancy resulted in higher total GWG and a significantly higher prevalence of SGA, caesarean section and HDP. This result was consistent with previous findings regarding a slow initial GWG but a later drastic GWG resulting in excessive GWG and a higher risk of caesarean section (Yong et al., 2020). Women in this group were younger and had a higher prepregnancy weight with a higher proportion of overweight and obesity and excessive GWG than the other two trajectories. Previous studies reported that younger women and overweight women were more likely to gain excessive GWG (Restall et al., 2014; Rodrigues et al., 2010; Samura et al., 2016; Yong et al., 2016). The younger mother may be likely to be pregnant for the first time, and most of them have poor knowledge of the consequence of excessive GWG, perceiving that higher GWG is beneficial for the baby health. While older mothers may have more experience with pregnancy and knowledge of GWG recommendations and thus tend to gain adequate GWG. This finding suggests that younger and overweight women should be the target for intervention to prevent excessive GWG and achieve healthy pregnancy outcomes.

Women in Trajectory 2 were the eldest and had the lowest proportion of prepregnancy underweight in the three trajectories, with the highest GWG in the first and second trimester (median GWG: 1.6 and 8.2 kg) and the lowest GWG in the third trimester (median GWG: 3.9 kg). Interestingly, there were two contrary patterns of GWG and maternal characteristics discovered by our latent class trajectory model. Women in Trajectory 2 had first high GWG and then low GWG, resulting in relatively lower GWG but significantly higher risk of LGA and lower risk of HDP, while women in Trajectory 3 showed lower GWG in early pregnancy and relatively higher GWG in mid and late pregnancy, resulting higher total GWG and higher risk of SGA, LBW, preterm birth, caesarean section and HDP but not significantly higher risk of LGA. These contradictory patterns and timing of GWG lead to apposite results. The catch‐up GWG pattern resulting in a significantly higher total GWG of Trajectory 3 did not affect the risk of LGA, which was consistent with previous findings (Gaillard et al., 2015; Zheng et al., 2019). In addition, this also showed the importance of early‐ and mid‐pregnancy weight gain on birthweight. The Agency for Health Care Research and Quality (AHRQ) evaluation also showed that increases in unit GWG during the first or second trimester have a stronger effect on birthweight than during the third trimester (Viswanathan et al., 2008). Similar results were obtained by previous research with positive associations between first‐ and second‐trimester GWG and infant birthweight (Abrams, 1995; Brown et al., 2002; Widen et al., 2015). A prospective cohort study established in China, which also evaluated the association of the timing of weight gain during pregnancy with infant birthweight, showed that only the maternal weight gain in the first half of gestation is a key determinant of infant birthweight (Retnakaran et al., 2018). The mechanisms of higher GWG in early pregnancy related to fetal overgrowth may be explained as serious insulin resistance programme or fetus‐related growth factor. For example, Rifas‐Shiman et al. (2017) confirmed that a higher GWG rate in the first trimester was associated with higher insulin levels and lower adiponectin levels in cord blood, while a higher GWG rate in the second trimester was associated with higher IGF and leptin levels in cord blood. IGF1 and leptin closely correlate with fetal size at birth (Donnelly et al., 2015; Inoue et al., 2008; Karakosta et al., 2013; Wiley et al., 2016). GWG may affect fetal glucose and insulin regulation and metabolism; there may be a critical window period for the effects of GWG in the first and second trimesters on fetal growth, although the underlying mechanisms warrant further exploration.

Women in Trajectory 3 had a greater GWG in the third trimester, a greater total GWG and a higher proportion of prepregnancy overweight and obesity, compared with women in Trajectory 2. Several studies have shown that women with greater GWG are more likely to develop HDP (Heude et al., 2012; Macdonald‐Wallis et al., 2013; Ren et al., 2018), as well as women with GDM (Barquiel et al., 2014). The results of an association between HDP and weight gain in the third trimester were reported by Gonzalez‐Ballano (2021). In that study, an increase risk of HDP with GWG in the third trimester was found. For Trajectory 2, we observed lower risk of HDP than Trajectory 1 in our study. Song et al. suggested that the rapid weight gain after 20 weeks may be a clinical warning sign of HDP in obese women (Song et al., 2023), and the low GWG in late pregnancy of Trajectory 2 may contribute to the low risk of HDP. HDP is possibly driven by vascular factors related to maternal fluid expansion and leads to caesarean section and poor fetal growth. Further research are needed to clarify these mechanisms. Our findings indicated that the reduction of HDP risk could be achieved by optimising total GWG and in the third trimester. Intervention may have the potential to prevent HDP, and future randomised trials should be conducted to assess the effectiveness of weight management during pregnancy in the reduction of HDP. The current study also found that the catch‐up GWG in trajectory also increased the risk of caesarean section and preterm birth, possibly because of the higher proportion of overweight and obesity. Overweight and obese women are more likely to develop caesarean section and preterm birth (Chen et al., 2020; Nkoka et al., 2019; Wang et al., 2019).

After stratification of the data, among overweight or obese women, the significant association between Trajectory 2 and LGA only remained among normal weight women. Meanwhile, the risk of SGA in the catch‐up GWG pattern was observed among all prepregnancy BMI categories. It's worth noting that the risks of GDM‐related adverse pregnancy outcomes in the normal weight group were more prominent than those in the overweight and obese group. The physiological differences caused by glucose metabolism may help explain the unexpected finding. An unhealthy intrauterine environment including hyperinsulinism and insulin resistance of GDM caused by prepregnancy overweight or obesity may attenuate some of that of high GWG in late pregnancy and probably help explain the negative result in the overweight or obese group. Given the results of the stratified analysis, our findings proved that this catch‐up pattern of weight gain should be even less desirable among normal weight women. Our results showed that uniform weight gain appeared to have the best pregnancy outcome. Furthermore, the observed adverse pregnancy outcomes by GWG trajectory were more pronounced by normal weight and overweight or obese women. Similarly, adverse pregnancy outcomes were more likely to occur among pregnant women who gained too much weight during pregnancy, especially if the pattern of weight gain follows the pattern of Trajectory 3. That is, this catch‐up GWG pattern has a higher incidence of adverse pregnancy outcomes in the context of excessive GWG than in the population of pregnant women with inadequate and adequate GWG. Weekly weight monitoring is necessary and can be corrected when weight gain exceeds the standard.

Weight management in GDM has been a research hotspot recently and studies mainly focus on the total GWG on pregnancy outcomes, rarely studies exploring GWG trajectories. Therefore, we identified the GWG trajectory by utilising a longitudinal approach and assessed risks using trajectories as independent factors. Clinical guidance is an important component of medical nutrition therapy for GDM, and our findings help obstetric clinicians accurately monitor weekly maternal weight gain to reach optimal and uniform weight gain and improve pregnancy outcomes. This is the first study to identify the GWG trajectories in Southeast China among GDM women. However, several limitations should be noted. Firstly, because of the retrospective study, some confounding factors such as family income and other factors that may influence the results could not be explained. Secondly, the prepregnancy BMI was calculated based on self‐reported weight before pregnancy, and weight measurement before 11 gestational weeks was not included as few women had antenatal visits before 11 gestational weeks. Since GWG in different trimesters is not an independent factor, GWG in the second or third trimesters might be influenced by weight gain in the previous trimester, and these may cause bias and limitations on the results. Thirdly, although we chose the first trajectory as a reference group, it was not clear whether it was the ‘best’ trajectory for women with GDM.

## 5 CONCLUSIONS

In summary, more than half of the GDM women in the study did not meet the Chinese weight gain guidelines. We identified three trajectories of GWG in GDM and evaluated the relationship between GWG trajectories and GDM‐related adverse pregnancy outcomes. Most of the GDM women in the current study had sustained moderate GWG throughout pregnancy. Women with a slow initial GWG but followed by drastic GWG had higher risks of adverse pregnancy outcomes. The observed adverse pregnancy outcomes by GWG trajectory were more pronounced by normal weight and overweight or obese women. Early recognition of an unhealthy GWG trajectory may contribute to early intervention in high‐risk groups to minimise adverse outcomes. Further research is needed to determine the ‘optimal’ trajectory and establish weekly GWG rate recommendations for the GDM population.

## AUTHOR CONTRIBUTIONS

Juan Lin and Jianying Yan designed the study and revised the manuscript. Yanhong Huang collected data, researched data and reviewed the manuscript. Lijuan Chen collected data, researched data and contributed to discussion. Lianghui Zheng and Yebin Feng revised and edited the manuscript. Lihua Lin was involved in the data analysis, manuscript drafting and manuscript revision. All authors approved the final version of the manuscript.

## CONFLICT OF INTEREST STATEMENT

The authors declare no conflict of interest.

## Supporting information

Supporting information.

## Data Availability

The data sets used and/or analysed during the current study are available from the corresponding authors on reasonable request.
