# Supplementary material for: Gestational weight trajectory and risk of adverse pregnancy outcomes among women with gestational diabetes mellitus: A retrospective cohort study
Source: Matern Child Nutr. 2024 Mar 22;20(3):e13645. doi: 10.1111/mcn.13645 (PMC11168372; doi:10.1111/mcn.13645)
Supplement: Supplementary file 1 — Supporting information. [file MCN-20-e13645-s001.docx]

Supplementary table 1 The Chinese gestational weight gain(GWG) recommendations.

| Pre-pregnancy body mass index | Total GWG range(kg) | Weekly GWG rate in the second and third trimester(kg) |
| --- | --- | --- |
| Underweight(BMI<18.5kg/m^2^) | 11.0~16.0 | 0.46(0.37~0.56) |
| Normal weight(18.5kg/m^2^≤BMI<24.0kg/m^2^) | 8.0~14.0 | 0.37(0.26~0.48) |
| Overweight(24.0kg/m^2^≤BMI<28.0kg/m^2^) | 7~11.0 | 0.30(0.22~0.37) |
| Obesity(BMI≥28.0kg/m^2^) | 5.0~9.0 | 0.22(0.15~0.30) |
